# Comparison of Plastic, Fully Covered Metal, and Intraductal Metal Stents for Biliary Anastomotic Strictures After Living Donor Liver Transplantation: A Single-Center Experience

**DOI:** 10.3390/jcm15145641

**Published:** 2026-07-18

**Authors:** Ömer Küçükdemirci, Berk Baş

**Affiliations:** 1 Department of Gastroenterology and Hepatology, School of Medicine, Ondokuz Mayıs University, 55100 Samsun, Türkiye; drkucukdemirci@yahoo.com; 2 Department of Gastroenterology and Hepatology, School of Medicine, Aydın Adnan Menderes University, 09100 Aydın, Türkiye

**Keywords:** liver transplantation, living donor liver transplantation, biliary anastomotic stricture, endoscopic retrograde cholangiopancreatography, fully covered self-expandable metal stent, intraductal metal stent, plastic stent

## Abstract

**Background**: Benign biliary anastomotic strictures (BAS) remain among the most frequent complications after living donor liver transplantation (LDLT). Although multiple plastic stents (MPS) have traditionally been considered the standard endoscopic treatment, fully covered self-expandable metal stents (FCSEMS) and intraductal fully covered self-expandable metal stents (ID-FCSEMS) have emerged as alternative strategies. This study aimed to compare the efficacy and safety of MPS, FCSEMS, and ID-FCSEMS in the management of post-transplant biliary anastomotic strictures. **Methods**: This retrospective single-center cohort study included consecutive adult patients who developed benign biliary anastomotic strictures after LDLT and underwent ERCP-based treatment between January 2020 and January 2025. Patients were categorized according to the initial stent strategy: MPS (*n* = 32), FCSEMS (*n* = 30), and ID-FCSEMS (*n* = 25). Primary outcome was overall treatment success. Secondary outcomes included recurrence, number of ERCP sessions, treatment duration, post-procedural hospitalization, stent migration, and procedure-related adverse events. **Results**: A total of 87 patients were included. Clinical success was achieved in 81.3% of patients treated with MPS, 83.3% treated with FCSEMS, and 92.0% treated with ID-FCSEMS (*p* = 0.501). Recurrence rates were 18.8%, 10.0%, and 12.0%, respectively (*p* = 0.579). Patients treated with metal stents required significantly fewer ERCP sessions than those treated with MPS (median 5 vs. 2 vs. 2; *p* < 0.001). Median treatment duration was significantly shorter in the FCSEMS and ID-FCSEMS groups compared with the MPS group (12 vs. 10 vs. 9 months; *p* < 0.001). Post-procedural hospitalization was also reduced with metal stents (7 vs. 4 vs. 3 days; *p* < 0.001). Adverse event rates, including cholangitis, pancreatitis, bleeding, perforation, and mortality, were comparable among groups. Stent migration occurred in 21.9% of MPS patients, 23.3% of FCSEMS patients, and 12.0% of ID-FCSEMS patients (*p* = 0.525). On multivariable analysis, MELD score was the only independent predictor of treatment success (adjusted OR 0.90, 95% CI 0.83–0.98; *p* = 0.012). **Conclusions**: MPS, FCSEMS, and ID-FCSEMS achieved comparable rates of stricture resolution, recurrence, and adverse events in the treatment of biliary anastomotic strictures after LDLT. However, FCSEMS and ID-FCSEMS significantly reduced the number of ERCP procedures, treatment duration, and hospitalization compared with conventional plastic stenting. Among the evaluated strategies, ID-FCSEMS demonstrated the highest numerical success rate and lowest migration rate, suggesting a potential procedural advantage while maintaining comparable safety and efficacy.

## 1. Introduction

Benign biliary anastomotic strictures (BAS) remain one of the most frequent biliary complications after liver transplantation, with reported incidences ranging between 5% and 40%, particularly following living donor liver transplantation (LDLT) because of smaller duct diameter, multiple bile duct anastomoses, and technically complex biliary reconstruction [1,2]. These strictures may lead to recurrent cholangitis, graft dysfunction, repeated hospitalizations, impaired quality of life, and, in severe cases, graft loss or the need for retransplantation [3,4]. Early recognition and effective endoscopic management are therefore critical to preserve long-term graft and patient outcomes.

Endoscopic retrograde cholangiopancreatography (ERCP) is currently accepted as the first-line therapeutic modality for post-transplant biliary anastomotic strictures in patients with duct-to-duct reconstruction because it provides both diagnostic evaluation and minimally invasive treatment in the same session [5,6]. Over the past two decades, advances in endoscopic accessories and stent technology have substantially improved treatment outcomes, and ERCP-based management has largely replaced percutaneous and surgical approaches in most transplant centers [7]. Nevertheless, despite high technical success rates, the optimal endoscopic stent strategy for benign post-transplant biliary strictures remains controversial.

Traditionally, sequential placement of multiple plastic stents (MPS) has been considered the standard endoscopic treatment approach for benign biliary anastomotic strictures after liver transplantation [8]. This strategy is based on progressive stricture dilation achieved through repeated ERCP sessions with increasing numbers and diameters of plastic stents over time. Reported long-term stricture resolution rates with MPS therapy generally range between 80% and 95% [9,10]. Historically, sequential placement of multiple plastic stents has been considered the standard endoscopic treatment approach for post-transplant biliary anastomotic strictures and has been widely adopted in clinical practice owing to its high long-term stricture resolution rates [11]. However, this approach requires multiple ERCP procedures over prolonged treatment periods, often every 2–3 months, resulting in increased procedural burden, higher healthcare costs, cumulative anesthesia exposure, and reduced patient comfort [12,13]. In addition, repeated interventions may increase the risk of procedure-related adverse events such as cholangitis, pancreatitis, bleeding, and stent occlusion [14].

In recent years, fully covered self-expandable metal stents (FCSEMS) have emerged as an alternative treatment option for post-transplant biliary strictures [15]. Owing to their larger diameter and greater radial expansion force, FCSEMS may achieve more rapid stricture remodeling while reducing the number of required ERCP sessions and shortening overall treatment duration [16]. Several comparative studies and meta-analyses have suggested that FCSEMS provide similar or superior stricture resolution rates compared with multiple plastic stents, with fewer endoscopic procedures and shorter indwelling periods [17,18]. These potential advantages have led to increasing adoption of FCSEMS in routine transplant endoscopy practice.

Despite these benefits, conventional transpapillary FCSEMS are associated with important limitations. Stent migration remains one of the most frequently reported complications, with migration rates reaching up to 30% in some series [19]. In addition, coverage of the pancreatic duct orifice by long transpapillary metal stents may increase the risk of post-ERCP pancreatitis, particularly in patients without prior sphincterotomy [20]. Other concerns include sludge formation, cholangitis, tissue hyperplasia at stent ends, and difficulties related to stent removal after prolonged indwelling time [21]. As a result, the ideal metal stent design for benign post-transplant biliary strictures has not yet been clearly established.

To overcome these limitations, novel intraductal fully covered metal stents with transpapillary extraction strings have recently been introduced [22]. Unlike conventional FCSEMS, these shorter intraductal stents are positioned entirely above the papilla, thereby minimizing direct compression of the pancreatic orifice and potentially reducing the risk of pancreatitis and distal migration [23]. The attached retrieval string extending into the duodenum facilitates easy endoscopic removal while preserving the advantages of metal stent radial force and prolonged patency [24]. Preliminary studies evaluating intraductal FCSEMS in benign biliary strictures have reported encouraging outcomes, including high stricture resolution rates, lower migration rates, and fewer required ERCP sessions [25,26]. However, data specifically focusing on LDLT-associated biliary anastomotic strictures remain limited, and direct comparisons between sequential plastic stents, conventional FCSEMS, and intraductal FC.

## 2. Methods

### 2.1. Study Design and Patient Selection

This retrospective single-center cohort study was conducted at the Aydın University Organ Transplantation Center, a tertiary referral center for liver transplantation and advanced biliary endoscopy. Consecutive adult patients who developed benign biliary anastomotic strictures after liver transplantation and underwent endoscopic retrograde cholangiopancreatography (ERCP)-based treatment between January 2020 and December 2023 were retrospectively evaluated. During the study period, a total of 428 living donor liver transplantations were performed at our institution, and biliary anastomotic strictures developed in 107 recipients (25.0%). Eighty-seven patients who underwent definitive conventional ERCP-based stent therapy met the eligibility criteria for the present comparative analysis, whereas 20 patients received alternative rescue interventions and were excluded as described below.

The included patients were categorized into three groups according to the initial endoscopic stent strategy: sequential multiple plastic stents (MPS; Group 1, n = 32), conventional fully covered self-expandable metal stents (FCSEMS; Group 2, n = 30), and intraductal fully covered self-expandable metal stents (ID-FCSEMS; Group 3, n = 25). Stent selection was based on biliary anatomy, stricture characteristics, technical feasibility, previous endoscopic interventions, and endoscopist preference according to evolving institutional treatment protocols during the study period.

Benign biliary anastomotic strictures were diagnosed on the basis of clinical symptoms, cholestatic liver enzyme abnormalities, and imaging findings obtained by magnetic resonance cholangiopancreatography (MRCP) and/or ERCP. Anastomotic strictures were defined as localized narrowing confined to the biliary anastomosis without radiological evidence of diffuse intrahepatic biliary involvement.

The primary outcome was overall treatment success, defined as achievement of both technical and clinical success with subsequent radiological resolution of the stricture during follow-up. Technical success was defined as successful biliary cannulation, fluoroscopic traversal of the stricture, and successful deployment of the intended stent. Clinical success was defined as improvement in cholestatic liver function tests together with resolution of biliary obstruction-related symptoms after endoscopic treatment. Secondary outcomes included the number of ERCP sessions, treatment duration, recurrence, stent migration, post-ERCP pancreatitis, cholangitis, bleeding, and the need for percutaneous or surgical intervention.

Inclusion criteria were age ≥ 18 years, liver transplantation with duct-to-duct biliary reconstruction, benign biliary anastomotic stricture confirmed by clinical, laboratory, and imaging findings, ERCP-based treatment using plastic or fully covered metal stents. Consecutive adult patients who developed benign biliary anastomotic strictures after living donor liver transplantation and underwent ERCP-based treatment between January 2020 and December 2023 were retrospectively evaluated. All patients were followed for a minimum of 24 months after the index ERCP procedure. Follow-up was completed through December 2025, and only patients with complete follow-up data were included in the final analysis.

Patients were excluded if they had malignant biliary strictures, pre-existing biliary strictures before transplantation, non-anastomotic strictures including ischemic cholangiopathy or diffuse intrahepatic strictures, primary treatment modalities other than ERCP, combined use of plastic and metal stents during the same treatment period, insufficient follow-up data, or contraindications to ERCP due to severe coagulopathy or surgically altered anatomy preventing endoscopic access. Patients who did not undergo definitive conventional ERCP-based stent therapy were also excluded. This included patients who remained dependent on definitive percutaneous transhepatic biliary drainage (PTBD) because the anastomotic stricture could not be traversed endoscopically, those treated with magnetic compression anastomosis, and those managed using a combined percutaneous-endoscopic rendezvous technique after failed conventional ERCP, as these represent fundamentally different therapeutic strategies that would preclude a valid comparison with conventional ERCP-based stent treatments. However, patients who underwent temporary bridging PTBD followed by successful definitive ERCP-based treatment were included in the study cohort.

Clinical, laboratory, transplant-related, and procedural data were retrospectively collected from the institutional electronic medical record system and endoscopy database. The study protocol was approved by the institutional ethics committee and conducted in accordance with the ethical principles of the Declaration of Helsinki.

### 2.2. Endoscopic Procedure, Definitions, and Follow-Up

All ERCP procedures were performed by experienced therapeutic endoscopists, each of whom had independently performed more than 1000 ERCP procedures, using a therapeutic side-viewing duodenoscope (Fujifilm/Fujinon ED-580XT series, Fujifilm Corp., Tokyo, Japan). Procedures were carried out under deep sedation or general anesthesia according to patient condition and anesthesiology evaluation. All patients received prophylactic broad-spectrum intravenous antibiotics before ERCP in accordance with current transplant endoscopy guidelines. In addition, rectal diclofenac (100 mg) was routinely administered for prophylaxis against post-ERCP pancreatitis unless contraindicated.

Selective biliary cannulation was achieved using a standard sphincterotome and guidewire-assisted technique. Following cholangiographic confirmation of the biliary anastomotic stricture, endoscopic sphincterotomy was performed in all patients. Stricture characteristics were systematically evaluated during ERCP using fluoroscopic cholangiography before therapeutic intervention. The location, length, degree of luminal narrowing, and proximal biliary dilatation were assessed in all patients. Stricture length was determined by measuring the maximal longitudinal extent of the narrowed segment on fluoroscopic cholangiography and was categorized as short, intermediate, or long according to its longitudinal extent. Stricture severity was determined by comparing the luminal diameter at the narrowest point of the stricture with the diameter of the adjacent normal bile duct. Mild strictures were defined as <50% reduction in luminal diameter, moderate strictures as 50–75% reduction, and severe strictures as >75% reduction. All fluoroscopic assessments were performed independently by two experienced therapeutic endoscopists at the time of ERCP, and disagreements were resolved by consensus. In addition, time from liver transplantation to stricture diagnosis, bile duct anastomosis type, previous bile leak, previous ERCP, and proximal biliary dilatation were recorded for all patients and analyzed as baseline stricture-related and procedural characteristics. Balloon dilation was generally performed using controlled radial expansion (CRE) balloons ranging from 4 to 8 mm according to bile duct diameter and stricture severity.

In the MPS group, plastic biliary stents measuring 7–11.5 Fr were positioned across the anastomotic stricture with progressive upsizing and increasing stent numbers during scheduled ERCP sessions at approximately 10–12-week intervals. Plastic stents from Boston Scientific (Marlborough, MA, USA), Cook Medical (Bloomington, IN, USA), and Micro-Tech (Nanjing, China) were used according to availability and biliary anatomy.

In the FCSEMS group, fully covered metal stents measuring 8–10 mm in diameter and 40–80 mm in length were deployed transpapillary across the stricture under fluoroscopic guidance. FCSEMS devices were primarily obtained from Boston Scientific and Micro-Tech and were generally left in situ for 6–9 months unless earlier removal was required because of migration, cholangitis, or stent occlusion.

In the ID-FCSEMS group, short fully covered intraductal metal stents (Micro-Tech, Nanjing, China) equipped with a transpapillary retrieval string were positioned entirely above the papilla, while the retrieval string was left extending into the duodenal lumen to facilitate endoscopic removal. These stents were selected to minimize papillary compression and reduce the risks of post-ERCP pancreatitis and distal migration while preserving prolonged stent patency and radial expansion force. Intraductal FCSEMS were generally removed after approximately 6 months if complete stricture resolution was achieved.

Patients were followed clinically, biochemically, and radiologically after ERCP. Additional ERCP sessions were performed when clinically indicated because of persistent cholestasis, recurrent symptoms, stent dysfunction, or radiological suspicion of residual or recurrent stricture.

Stricture resolution was defined as absence of significant residual narrowing on follow-up cholangiography together with normalization or marked improvement in cholestatic biochemical parameters after scheduled stent removal. Treatment duration was defined as the interval between the initial ERCP session and documented stricture resolution with final stent removal. Recurrence was defined as redevelopment of symptomatic or biochemical biliary obstruction associated with radiological evidence of recurrent anastomotic narrowing after initial successful treatment. Stent migration was defined as partial or complete displacement of the stent from its intended position confirmed fluoroscopically or endoscopically during follow-up.

Procedure-related adverse events were evaluated according to established consensus criteria. Post-ERCP pancreatitis was defined according to the revised Atlanta criteria. Cholangitis was defined according to the Tokyo Guidelines criteria. Post-procedural bleeding was defined as clinically significant bleeding requiring transfusion, endoscopic hemostatic therapy, angiographic intervention, or prolonged hospitalization.

### 2.3. Statistical Analysis

Continuous variables were expressed as mean ± standard deviation (SD) or median with interquartile range (IQR), depending on data distribution. Categorical variables were presented as frequencies and percentages. Normality of continuous variables was assessed using appropriate tests of distribution. Comparisons among treatment groups were performed using one-way analysis of variance (ANOVA) or the Kruskal–Wallis test for continuous variables, as appropriate, and the chi-square test or Fisher’s exact test for categorical variables. Predictors of clinical success were initially evaluated using univariable logistic regression analysis. Subsequently, multivariable logistic regression analysis was performed to identify independent predictors of clinical success. Because recipient age and MELD score differed significantly among treatment groups at baseline, additional adjusted analyses were performed to account for these potential confounders. Binary outcomes (clinical success, recurrence, and stent migration) were analyzed using multivariable logistic regression, whereas continuous outcomes (number of ERCP sessions, treatment duration, post-procedural hospital stay, and follow-up duration) were analyzed using multivariable linear regression. Recipient age and MELD score were included as covariates in all adjusted models. A two-sided *p* value < 0.05 was considered statistically significant. Statistical analyses were performed using IBM SPSS Statistics for Windows, version 29.0 (IBM Corp., Armonk, NY, USA).

### 2.4. Ethical Approval

The study was approved by the Aydın University Clinical Research Ethics Committee (Approval No: 2026/20, Date: 21 January 2026). All procedures were conducted in accordance with the Declaration of Helsinki.

Use of Generative Artificial Intelligence (GenAI): ChatGPT-5.5 (OpenAI, San Francisco, CA, USA) was used solely for language editing, grammar correction, and improvement in manuscript readability during manuscript preparation. The AI tool was not used for data collection, statistical analysis, interpretation of results, or generation of scientific conclusions. All scientific content and final manuscript revisions were reviewed and approved by the authors.

## 3. Results

During the study period, 428 living donor liver transplantations were performed at our institution, of which 107 recipients (25.0%) developed biliary anastomotic strictures. Of these, 87 patients who underwent definitive conventional ERCP-based stent therapy fulfilled the study eligibility criteria and were included in the final comparative analysis. According to the initial stent strategy, 32 patients were treated with sequential multiple plastic stents, 30 with conventional fully covered self-expandable metal stents (FCSEMS), and 25 with intraductal fully covered metal stents (ID-FCSEMS).

An additional 20 patients with biliary anastomotic strictures fulfilled predefined exclusion criteria and were therefore not included in the final analysis. These comprised six patients who remained dependent on definitive percutaneous transhepatic biliary drainage (PTBD) because the anastomotic stricture could not be traversed endoscopically, eight patients treated with magnetic compression anastomosis, and six patients managed using a combined percutaneous-endoscopic rendezvous technique following failed conventional ERCP. These patients were excluded because the primary objective of the present study was to compare outcomes among patients undergoing conventional ERCP-based stent treatment strategies.

Baseline demographic and transplant-related characteristics are summarized in Table 1. Significant differences were observed between groups with respect to age and MELD score. Patients treated with intraductal metal stents were older than those in the plastic and FCSEMS groups (58.6 vs. 46.2 and 55.2 years, respectively; *p* < 0.001). Conversely, patients in the plastic stent group had significantly higher MELD scores at the time of transplantation compared with the FCSEMS and intraductal metal stent groups (19.3 vs. 14.6 and 15.0, respectively; *p* = 0.02).

Male sex predominated in all treatment groups, accounting for 62.5% of patients in the plastic stent group, 46.7% in the FCSEMS group, and 48.0% in the intraductal metal stent group; however, this difference was not statistically significant (*p* = 0.39). The vast majority of transplantations were performed using right-lobe grafts, with proportions exceeding 90% in all groups. Left-lobe grafts were uncommon and showed no significant difference among treatment strategies.

No patient developed hepatic artery thrombosis. Portal vein complications were infrequent and occurred in only four patients overall, with no significant intergroup differences. Similarly, cold ischemia time, duration of post-transplant hospitalization, and the need for PTK-assisted initial biliary cannulation were comparable among groups. These findings suggest that, apart from age and MELD score, baseline demographic and transplant-related characteristics were generally well balanced across the three treatment strategies.

The underlying indications for liver transplantation are summarized in Table 2. Viral liver disease was the most common indication overall, accounting for 35 patients (40.2%), followed by cryptogenic cirrhosis in 18 patients (20.7%) and MASLD/NASH-related liver disease in 15 patients (17.2%). Alcohol-related liver disease and autoimmune liver diseases each accounted for 5.7% of transplant indications, while other less common etiologies represented 10.3% of cases.

Stricture-related and procedural characteristics are summarized in Table 3. The median time from liver transplantation to stricture diagnosis was comparable among the three treatment groups. Likewise, bile duct anastomosis type, stricture length, upstream biliary dilatation, previous bile leak, and previous ERCP did not differ significantly between groups. Stricture severity differed significantly among treatment groups (*p* = 0.044), whereas treatment duration (stent dwell time) differed as expected according to the endoscopic treatment strategy employed.

Across treatment groups, viral liver disease remained the predominant indication, comprising 34.4% of patients in the plastic stent group, 40.0% in the FCSEMS group, and 48.0% in the intraductal metal stent group. Cryptogenic cirrhosis was the second-most common etiology in the plastic and FCSEMS groups, whereas MASLD/NASH-related liver disease was relatively more frequent in the intraductal metal stent group. Alcohol-related and autoimmune liver diseases were infrequent across all groups. Overall, the distribution of liver transplantation etiologies appeared broadly comparable among the three treatment groups, without a clear predominance of any specific underlying liver disease that could account for differences in endoscopic outcomes.

Technical and clinical success rates were high across all treatment groups. Successful stricture resolution without the need for alternative biliary drainage modalities was achieved in 26 of 32 patients (81.3%) treated with plastic stents, 25 of 30 patients (83.3%) treated with FCSEMS, and 23 of 25 patients (92.0%) treated with intraductal metal stents. Although numerically higher success rates were observed in the intraductal metal stent group, the difference did not reach statistical significance (*p* = 0.501).

Stricture recurrence after initial successful treatment was observed in all groups. Recurrence occurred in six patients (18.8%) in the plastic stent group, three patients (10.0%) in the FCSEMS group, and three patients (12.0%) in the intraductal metal stent group. While recurrence appeared less frequent in patients treated with metal stents, these differences were not statistically significant (*p* = 0.579).

A marked difference was observed in the number of ERCP procedures required to achieve treatment completion. Patients treated with sequential plastic stenting underwent a median of five ERCP sessions, whereas those treated with FCSEMS or intraductal metal stents required only two sessions. This reduction represented one of the most notable advantages of metal stent strategies and reached strong statistical significance (*p* < 0.001).

Similarly, time to complete stricture resolution differed significantly among groups. Patients treated with plastic stents required a median treatment duration of 12 months, compared with 10 months in the FCSEMS group and 9 months in the intraductal metal stent group (*p* < 0.001). Thus, both metal stent approaches achieved stricture resolution more rapidly than conventional plastic stenting.

Post-procedural hospitalization also favored metal stent strategies. Because hospital stay was calculated as the cumulative duration of hospitalization throughout the entire endoscopic treatment course rather than after a single ERCP session, longer hospitalization in the MPS group was expected, given the need for repeated scheduled ERCP procedures and additional admissions for serial plastic stent exchanges. The median hospital stay following ERCP was 7 days in the plastic stent group, compared with 4 days in the FCSEMS group and only 3 days in the intraductal metal stent group (*p* < 0.001). These findings indicate that metal stents may reduce healthcare utilization and overall treatment burden compared with conventional plastic stenting (Table 4).

The incidence of procedure-related adverse events is shown in Table 5. Overall complication rates were comparable among treatment groups, and no significant differences were identified for any major complication.

Cholangitis was the most common adverse event, occurring in 12 patients (37.5%) in the plastic stent group, 10 patients (33.3%) in the FCSEMS group, and 8 patients (32.0%) in the intraductal metal stent group (*p* = 0.898). Despite numerical differences, the risk of cholangitis remained similar regardless of stent type.

Post-ERCP pancreatitis occurred infrequently and at comparable rates among groups, affecting 6.3%, 6.7%, and 8.0% of patients in the plastic, FCSEMS, and intraductal metal stent groups, respectively (*p* = 0.965). All cases were managed conservatively without long-term sequelae.

Clinically significant bleeding was uncommon. Bleeding events occurred in two patients (6.3%) receiving plastic stents, three patients (10.0%) receiving FCSEMS, and one patient (4.0%) receiving intraductal metal stents (*p* = 0.671). Likewise, perforation was rare, with only a single case occurring in the intraductal metal stent group and no cases observed in the other groups (*p* = 0.285).

ICU admission was numerically more frequent among patients treated with plastic stents, occurring in 28.1% of patients compared with 10.0% and 12.0% in the FCSEMS and intraductal metal stent groups, respectively. However, this difference did not reach statistical significance (*p* = 0.120). Mortality rates were low overall, occurring in two patients in the plastic stent group and one patient in the FCSEMS group, while no deaths were recorded among patients treated with intraductal metal stents (*p* = 0.439).

Stent migration represented another important outcome. Migration occurred in approximately one-fifth of patients treated with plastic stents or FCSEMS (21.9% and 23.3%, respectively), whereas a lower migration rate was observed with intraductal metal stents (12.0%). Nevertheless, these differences were not statistically significant (*p* = 0.525).

Univariate logistic regression analysis was performed to identify factors associated with clinical success. Neither FCSEMS nor intraductal metal stent use demonstrated a statistically significant advantage over plastic stents. Compared with plastic stents, FCSEMS showed an odds ratio (OR) of 1.15 (95% CI: 0.31–4.27, *p* = 0.830), whereas intraductal metal stents demonstrated an OR of 2.65 (95% CI: 0.49–14.47, *p* = 0.259).

Male sex was associated with significantly higher odds of clinical success (OR 4.62, 95% CI: 1.17–18.20, *p* = 0.029). In contrast, increasing MELD score was associated with lower odds of successful treatment (OR 0.91, 95% CI: 0.84–0.97, *p* = 0.007). Right-lobe graft transplantation demonstrated a trend toward improved outcomes but did not achieve statistical significance (OR 6.55, 95% CI: 0.83–51.36, *p* = 0.074).

Variables meeting prespecified criteria were entered into a multivariable logistic regression model. After adjustment for potential confounders, MELD score remained the only independent predictor of clinical success. Each one-point increase in MELD score reduced the likelihood of successful endoscopic treatment by approximately 10% (adjusted OR 0.90, 95% CI: 0.83–0.98, *p* = 0.012). Neither FCSEMS nor intraductal metal stent placement independently influenced treatment success after adjustment (Table 6).

Because recipient age and MELD score differed significantly between treatment groups at baseline, additional adjusted analyses were performed to evaluate whether these differences influenced the observed outcomes. After adjustment for age and MELD score, no significant differences were observed between treatment groups regarding clinical success, recurrence, or stent migration. In contrast, both FCSEMS and ID-FCSEMS remained independently associated with significantly fewer ERCP sessions, shorter treatment duration, and shorter post-procedural hospital stay compared with MPS. Follow-up duration was comparable among treatment groups after adjustment (Table 7).

To further evaluate the potential confounding effect of stricture severity, additional sensitivity analyses were performed including stricture severity as an additional covariate. The reductions in ERCP sessions, treatment duration, and post-procedural hospital stay associated with both FCSEMS and ID-FCSEMS remained statistically significant after this additional adjustment (Table 8). Although severe strictures were independently associated with longer treatment duration, they were not significantly associated with the number of ERCP sessions or post-procedural hospital stay. These findings indicate that the procedural advantages observed with metal stent strategies were not explained solely by the higher proportion of severe strictures in the ID-FCSEMS group.

Taken together, all three stent strategies achieved high rates of clinical success and acceptable safety profiles. Although overall success, recurrence, and complication rates were comparable, both FCSEMS and intraductal metal stents substantially reduced the number of ERCP sessions required, shortened time to stricture resolution, and decreased post-procedure hospitalization compared with conventional plastic stenting. These procedural advantages persisted after adjustment for baseline differences in recipient age and MELD score. Among the metal stent strategies, intraductal metal stents demonstrated the highest numerical success rate, the shortest treatment duration, the shortest hospitalization period, and the lowest stent migration rate, although these advantages did not reach statistical significance. Overall, these findings suggest that metal stent-based approaches may provide meaningful procedural and healthcare utilization benefits while maintaining comparable efficacy and safety to traditional multiple plastic stent therapy.

## 4. Discussion

The conventional management of anastomotic strictures (ASs) has traditionally involved repeated balloon dilatation and placement of plastic biliary stents, with stent exchange performed approximately every 3 months until stricture resolution is achieved. However, this strategy remains controversial, as some authors advocate longer stent exchange intervals, whereas others favor balloon dilatation alone in an effort to reduce procedure-related complications. Overall, ERCP is considered an effective and first-line therapeutic approach for ASs. Nevertheless, these strategies often require multiple ERCP sessions and repeated hospital admissions, thereby increasing patient burden and exposure to ERCP-related adverse events. In addition, the limited diameter and small caliber of conventional plastic stents frequently necessitate the placement of multiple stents to achieve adequate biliary dilatation, which can be technically demanding.

The baseline characteristics of our cohort largely reflect the profile of contemporary living donor liver transplantation (LDLT) recipients. Viral liver disease represented the most common indication for transplantation, followed by cryptogenic cirrhosis and MASLD/NASH-related liver disease. This distribution is consistent with previous LDLT series from Asia and the Middle East, where hepatitis B–related cirrhosis continues to account for a substantial proportion of liver transplantations despite advances in antiviral therapy. In contrast, alcohol-related liver disease and MASLD have become increasingly dominant indications in Western deceased-donor transplant programs. The predominance of viral liver disease in our cohort therefore likely reflects regional epidemiological characteristics rather than selection bias. Importantly, the distribution of transplant etiologies appeared broadly comparable among the three stent groups, suggesting that differences in underlying liver disease were unlikely to have significantly influenced endoscopic outcomes. Previous studies evaluating endoscopic management of post-transplant biliary strictures have similarly reported no consistent relationship between the primary liver disease etiology and stricture resolution rates [27,28].

Although most baseline variables were well-balanced among groups, significant differences were observed in recipient age and MELD score. Patients treated with plastic stents had significantly higher MELD scores than those treated with FCSEMS or intraductal metal stents. MELD score is widely recognized as a surrogate marker of pre-transplant disease severity and has been associated with increased postoperative morbidity, infectious complications, prolonged hospitalization, and inferior graft-related outcomes after liver transplantation. Consistent with these observations, MELD score emerged as the only independent predictor of clinical success in our multivariable analysis, whereas stent type itself did not independently influence treatment success. This finding suggests that recipient-related factors and overall disease severity may have a greater impact on long-term stricture resolution than the specific endoscopic treatment strategy employed. Notably, each one-point increase in MELD score was associated with an approximately 10% reduction in the likelihood of successful treatment, highlighting the potential importance of recipient condition in determining therapeutic outcomes [29]. To account for this baseline imbalance, additional adjusted analyses were performed using recipient age and MELD score as covariates. After adjustment, no significant differences were observed among the treatment groups with respect to clinical success, recurrence, or stent migration, confirming that the overall efficacy of the three stent strategies remained comparable. In contrast, the reductions in ERCP sessions, treatment duration, and post-procedural hospitalization associated with FCSEMS and ID-FCSEMS persisted after adjustment. These findings indicate that the procedural advantages of metal stent strategies are unlikely to be explained solely by differences in baseline disease severity and instead appear to reflect the intrinsic benefits of the stent strategy itself.

Other transplant-related variables were remarkably similar across groups. More than 95% of recipients underwent right-lobe LDLT, reflecting current surgical practice in adult living donor transplantation, where right-lobe grafts are preferred because they provide adequate graft volume and improve graft-to-recipient weight ratio. Previous studies have suggested that graft type may influence the risk of biliary complications, particularly when technical complexity is increased in left-lobe grafts. However, because right-lobe grafts overwhelmingly predominated in all three groups, graft type was unlikely to act as a confounding factor in the present study. Likewise, cold ischemia times and post-transplant hospitalization durations were comparable between groups, indicating similar perioperative conditions at baseline [30,31].

Another noteworthy finding was the complete absence of hepatic artery thrombosis (HAT) in the study population. HAT is one of the most important vascular complications following liver transplantation and has been consistently identified as a major risk factor for ischemic biliary injury, biliary strictures, graft dysfunction, and graft loss. Because the biliary epithelium depends almost entirely on arterial blood supply, even minor arterial insufficiency may adversely affect biliary healing and increase stricture formation. Several previous studies have demonstrated a strong association between hepatic artery thrombosis and post-transplant biliary complications. Verdonk et al. [32]. reported that arterial insufficiency significantly increases the risk of ischemic cholangiopathy, recurrent strictures, and endoscopic treatment failure following liver transplantation. In this context, the complete absence of HAT in our cohort represents a favorable characteristic of the study population and minimizes one of the strongest confounding factors known to affect biliary outcomes after transplantation The absence of HAT in our cohort eliminates one of the strongest known confounders affecting biliary outcomes and allows a more direct comparison of stent-related treatment strategies. Similarly, portal vein complications were infrequent and evenly distributed among groups, while the need for PTK-assisted biliary cannulation was rare in all treatment arms. Taken together, these findings indicate that baseline recipient, graft, and perioperative characteristics were generally comparable among groups, thereby strengthening the validity of the observed comparisons between plastic stents, FCSEMS, and intraductal metal stents [33,34,35].

Cold ischemia times were similarly short across all groups and did not differ significantly. Prolonged cold ischemia has repeatedly been identified as a major contributor to ischemia–reperfusion injury and subsequent biliary complications after liver transplantation. Sanchez–Urdazpal et al., Feng et al., and Foley et al. demonstrated that increasing preservation times are associated with higher rates of ischemic cholangiopathy and biliary strictures, primarily through injury to the peribiliary vascular plexus [36,37,38]. In contrast, living donor liver transplantation is typically characterized by substantially shorter ischemia times than deceased donor transplantation. The relatively short cold ischemia times observed in our cohort likely minimized ischemia-related biliary injury and reduced its potential influence on treatment outcomes. Similarly, post-transplant hospitalization duration was comparable among groups, suggesting similar perioperative recovery and baseline clinical status. Collectively, these findings indicate that vascular, ischemic, and perioperative factors were generally balanced among groups, strengthening the validity of the observed comparisons between plastic stents, FCSEMS, and intraductal metal stents [39,40,41].

Another important strength of the present study was the systematic evaluation of stricture-related characteristics using standardized fluoroscopic criteria. Time from liver transplantation to stricture diagnosis, bile duct anastomosis type, stricture length, proximal biliary dilatation, previous bile leak, and previous ERCP were broadly comparable among treatment groups. Although stricture severity differed significantly between groups, additional adjusted analyses demonstrated that the reductions in ERCP sessions, treatment duration, and post-procedural hospitalization associated with FCSEMS and ID-FCSEMS persisted after adjustment for recipient age and MELD score. These findings suggest that the procedural advantages observed with metal stent strategies are unlikely to be explained solely by baseline differences in recipient characteristics or stricture morphology.

The principal finding of the present study is that although overall clinical success, recurrence rates, and complication profiles were comparable among treatment groups, metal stent-based strategies substantially reduced treatment burden compared with conventional multiple plastic stenting. Patients treated with FCSEMS or intraductal metal stents required significantly fewer ERCP sessions, achieved stricture resolution in a shorter period, and experienced shorter post-procedural hospitalization than those managed with sequential plastic stents. These findings suggest that the primary advantage of metal stents lies not necessarily in improving ultimate treatment success, but rather in streamlining the therapeutic course and reducing the number of interventions required to achieve stricture resolution [42,43].

The traditional endoscopic management of post-transplant anastomotic strictures relies on repeated balloon dilation and sequential placement of increasing numbers of plastic stents over several months. Although this strategy has demonstrated favorable long-term success rates, it necessitates multiple scheduled ERCP procedures to progressively enlarge the biliary lumen. Consequently, patients are exposed to repeated hospital admissions, procedural risks, anesthesia exposure, and increased healthcare utilization. In contrast, fully covered metal stents provide a substantially larger luminal diameter immediately after deployment and exert continuous radial expansion across the stricture. This sustained dilatory force may accelerate biliary remodeling and facilitate earlier stricture resolution, thereby reducing the need for repeated interventions [15,35].

Our findings are consistent with previous studies comparing plastic stents and FCSEMS for post-transplant biliary strictures. Martins et al. [30] demonstrated that FCSEMS achieved stricture resolution rates comparable to multiple plastic stents while significantly reducing the number of ERCP procedures required during treatment. Similarly, Kaffes et al. [35] reported shorter treatment duration and fewer endoscopic interventions with FCSEMS without compromising long-term efficacy. A recent meta-analysis further confirmed that metal stents are associated with a lower procedural burden while maintaining similar rates of stricture resolution and recurrence compared with conventional plastic stenting. The results of our study extend these observations to a predominantly living donor liver transplantation population [35,44,45,46].

One of the most clinically relevant findings of the present study was the marked reduction in ERCP sessions among patients treated with metal stents. The median number of procedures decreased from five in the plastic stent group to only two in both metal stent groups. From a patient perspective, this reduction translates into fewer hospital visits, less procedural discomfort, and reduced cumulative exposure to ERCP-related adverse events [22,43]. From a healthcare perspective, fewer interventions may also reduce overall treatment costs and resource utilization [46,47]. Given that long-term clinical success remained comparable among groups, the ability to achieve similar outcomes with substantially fewer procedures may represent a significant practical advantage of metal stent-based strategies. The reduction in the number of ERCP procedures was also reflected in cumulative hospitalization during treatment. Because cumulative hospitalization represents the total duration of hospitalization accumulated throughout the entire endoscopic treatment course rather than the length of stay following a single ERCP procedure, longer cumulative hospitalization was expected in the MPS group. Sequential plastic stenting typically requires scheduled ERCP sessions approximately every three months with repeated hospital admissions for serial stent exchanges, whereas FCSEMS and ID-FCSEMS generally require substantially fewer planned interventions. Therefore, the shorter cumulative hospitalization observed in the metal stent groups most likely reflects the inherent characteristics of the treatment strategy rather than increased disease severity or procedure-related complications.

The clinical success rates observed in our study are broadly consistent with those reported in the existing literature. Multiple plastic stent protocols have historically achieved stricture resolution rates ranging from approximately 75% to 95%, while FCSEMS studies have reported success rates between 70% and 100%, depending on patient selection, stent dwell time, and follow-up duration. Martins et al. [30] reported stricture resolution rates of approximately 83% with FCSEMS and 97% with multiple plastic stents, whereas Kaffes et al. [35] demonstrated successful treatment in nearly 80% of transplant recipients treated with FCSEMS. More recently, intraductal fully covered metal stents have achieved success rates ranging from 80% to over 90% in selected patient populations. The success rates observed in our cohort (81.3% for plastic stents, 83.3% for FCSEMS, and 92.0% for intraductal metal stents) therefore fall within the upper range of outcomes reported in previous studies [47,48,49].

Despite the favorable procedural outcomes observed with metal stents, neither FCSEMS nor intraductal metal stents demonstrated a statistically significant advantage in overall clinical success or recurrence rates compared with conventional plastic stenting. Although intraductal metal stents achieved the highest numerical success rate, the difference did not reach statistical significance. Several factors may explain this finding. First, endoscopic treatment of anastomotic biliary strictures after living donor liver transplantation is generally associated with high success rates regardless of stent type. Consequently, demonstrating superiority between treatment modalities becomes increasingly difficult when baseline efficacy is already high across all groups. Second, the relatively limited sample size may have reduced the statistical power required to detect modest differences in clinical success. While the absolute difference between the plastic stent and intraductal metal stent groups exceeded 10%, the number of patients included in each treatment arm may have been insufficient to establish statistical significance.

Similarly, recurrence rates in our study were comparable among groups and were consistent with those reported previously. Published recurrence rates following successful endoscopic treatment generally range between 10% and 30%, irrespective of stent type. In the present study, recurrence occurred in 18.8% of patients treated with plastic stents, 10.0% of those treated with FCSEMS, and 12.0% of those treated with intraductal metal stents. Although recurrence was numerically lower in the metal stent groups, these differences were not statistically significant. Collectively, these findings suggest that all three treatment strategies provide durable long-term stricture control, while the principal advantage of metal stents appears to be a reduction in treatment burden rather than a substantial improvement in ultimate efficacy [48,49].

An important finding of the present study was the comparable safety profile observed among the three treatment strategies. Rates of cholangitis, post-ERCP pancreatitis, bleeding, perforation, stent migration, ICU admission, and mortality did not differ significantly between the plastic stent, FCSEMS, and intraductal metal stent groups. Cholangitis represented the most common adverse event, occurring in approximately one-third of patients across all groups, whereas post-ERCP pancreatitis and bleeding were relatively infrequent. Only a single perforation was observed in the intraductal stent group, and no significant difference in overall adverse event rates was identified.

The complication rates observed in our cohort are generally consistent with those reported in previous studies evaluating endoscopic treatment of post-transplant biliary strictures. Cholangitis rates ranging from 15% to 40% have been reported following both plastic stent and FCSEMS placement, reflecting the susceptibility of transplant recipients to biliary infection during prolonged endoscopic treatment. Similarly, post-ERCP pancreatitis rates between 3% and 10% have been described in most comparative series, with no consistent evidence demonstrating a significant difference between plastic and metal stent strategies. Martins et al. [30] and Kaffes et al. [35] likewise reported comparable overall adverse event rates despite differences in stent design and treatment protocols [50].

One of the historical concerns regarding FCSEMS placement has been the potential risk of stent migration. Previous studies have reported migration rates ranging from approximately 10% to 35%, making migration one of the most frequently encountered stent-related adverse events. In our study, migration occurred in 21.9% of patients treated with plastic stents, 23.3% of those treated with FCSEMS, and 12.0% of those treated with intraductal metal stents. Although these differences did not reach statistical significance, the numerically lower migration rate observed in the intraductal stent group is noteworthy and may reflect the design characteristics of intraductal devices, which remain entirely within the bile duct and are therefore less exposed to distal displacement forces [51].

Bleeding and perforation rates in our cohort were also comparable to those reported previously. Most studies evaluating FCSEMS and plastic stents have described bleeding rates below 10% and perforation rates below 2–5%, indicating that both approaches can be performed safely in experienced centers. Likewise, mortality rates remained low across all groups and were not directly attributable to stent type. Collectively, these findings suggest that metal stent-based approaches provide the procedural advantages of fewer ERCP sessions and shorter treatment duration without increasing the risk of major adverse events. Therefore, concerns regarding safety should not represent a major barrier to the use of FCSEMS or intraductal metal stents in appropriately selected patients with post-transplant anastomotic biliary strictures [52,53].

One particularly noteworthy observation in the present study was the numerically lower stent migration rate observed in the intraductal metal stent group. Although the difference did not reach statistical significance, migration occurred in only 12.0% of patients treated with intraductal stents compared with 21.9% and 23.3% in the plastic stent and FCSEMS groups, respectively. Stent migration remains one of the most common limitations of conventional FCSEMS therapy and frequently necessitates additional endoscopic interventions, increases treatment complexity, and may contribute to treatment failure in selected cases.

The lower migration rate observed with intraductal metal stents is likely attributable to their unique design and deployment characteristics. Unlike conventional FCSEMS, intraductal stents are positioned entirely within the bile duct and do not extend across the papilla into the duodenum. As a result, they are less exposed to duodenal peristalsis, papillary movement, and distal traction forces that may contribute to stent displacement. Furthermore, preservation of the sphincter mechanism may reduce duodenobiliary reflux and improve stent stability during prolonged indwelling periods.

Our findings are consistent with previous studies evaluating intraductal fully covered metal stents in post-transplant biliary strictures. Tal et al. and subsequent investigators reported relatively low migration rates with intraductal devices, suggesting that the intraductal approach may overcome one of the major drawbacks of conventional FCSEMS therapy. In addition to lower migration rates, several studies have demonstrated favorable stricture resolution rates and acceptable safety profiles with intraductal stents, supporting their growing role in the management of post-transplant anastomotic strictures.

Interestingly, the lower migration rate in the intraductal stent group was accompanied by the highest numerical clinical success rate and the shortest time to stricture resolution observed in our cohort. Although the present study was not powered to establish a direct causal relationship between migration and treatment success, these findings raise the possibility that improved stent stability may contribute to more effective and sustained stricture remodeling. Larger prospective studies are required to determine whether the reduced migration associated with intraductal stents translates into superior long-term clinical outcomes. Nevertheless, our results support the concept that intraductal metal stents may provide a meaningful technical advantage over conventional FCSEMS while maintaining comparable efficacy and safety.

An important consideration when interpreting migration rates is that stent migration did not invariably result in treatment failure. In clinical practice, management following migration is individualized according to stricture characteristics, timing of migration, and endoscopic findings. In our cohort, some patients underwent repeat placement of the same stent type, whereas others were managed with an alternative stent strategy at the discretion of the treating endoscopist. Consequently, migration should be viewed as a treatment-related adverse event rather than a definitive indicator of therapeutic failure. This may partly explain why differences in migration rates were not accompanied by corresponding differences in overall clinical success. Similar approaches have been reported in previous studies of post-transplant biliary strictures, where successful stricture resolution could still be achieved despite interim stent migration following repeat endoscopic intervention.

An additional sensitivity analysis was performed to evaluate whether the higher proportion of severe strictures in the ID-FCSEMS group influenced the observed treatment outcomes. After further adjustment for stricture severity in addition to recipient age and MELD score, the reductions in ERCP sessions, treatment duration, and post-procedural hospital stay associated with FCSEMS and ID-FCSEMS remained statistically significant. As expected, severe strictures were independently associated with longer treatment duration, reflecting the greater complexity of these lesions and the need for prolonged endoscopic therapy. In contrast, stricture severity was not independently associated with the number of ERCP sessions or post-procedural hospital stay. These findings further support that the procedural advantages observed with metal stent strategies cannot be explained solely by baseline differences in stricture severity.

Several limitations of this study should be acknowledged. First, the retrospective design introduces the potential for selection bias and unmeasured confounding despite the inclusion of consecutive patients. Second, this was a single-center study, which may limit the generalizability of the findings to other transplant programs with different patient populations and endoscopic practices. Third, the relatively modest sample size may have limited the statistical power to detect small but potentially clinically relevant differences in clinical success, recurrence, and complication rates among treatment groups. Fourth, treatment allocation was not randomized and reflected evolving clinical practice patterns over the study period, introducing the possibility of temporal bias. Fifth, patients requiring definitive rescue therapies, including permanent percutaneous transhepatic biliary drainage (PTBD), magnetic compression anastomosis, or a combined percutaneous-endoscopic rendezvous technique, were intentionally excluded because these represent fundamentally different therapeutic approaches. Although this allowed a more homogeneous comparison of conventional ERCP-based stent strategies, it may limit the generalizability of our findings to patients with more complex biliary strictures requiring rescue interventions. Sixth, because intraductal FCSEMS represented a newer treatment option, their use became increasingly adopted during the latter part of the study period. Consequently, a temporal effect related to evolving treatment preferences and increasing familiarity with this device cannot be completely excluded. However, treatment allocation was determined primarily according to biliary anatomy, stricture characteristics, technical feasibility, previous endoscopic interventions, and endoscopist preference rather than chronological order alone. Therefore, although a degree of temporal confounding is possible, clinical decision-making remained principally driven by patient- and stricture-related factors. Finally, management following stent migration was individualized according to endoscopic findings and operator preference, which may have influenced long-term outcomes in a subset of patients.

Despite these limitations, the study has several notable strengths. To our knowledge, this is one of the few studies directly comparing sequential plastic stents, conventional FCSEMS, and intraductal FCSEMS in a predominantly living donor liver transplantation population. In addition, all patients were managed at a high-volume transplant center using standardized endoscopic techniques and follow-up protocols, allowing a relatively homogeneous comparison of contemporary treatment strategies for post-transplant anastomotic biliary strictures.

In conclusion, plastic stents, FCSEMS, and intraductal FCSEMS achieved comparable rates of clinical success, recurrence, and procedure-related adverse events in the treatment of post-transplant anastomotic biliary strictures following living donor liver transplantation. However, metal stent-based strategies were associated with significant reductions in the number of ERCP sessions, time to stricture resolution, and post-procedural hospitalization compared with conventional multiple plastic stenting.

Among the evaluated treatment options, intraductal FCSEMS demonstrated the highest numerical clinical success rate, the shortest treatment duration, and the lowest stent migration rate, while maintaining a safety profile comparable to the other strategies. Although these advantages did not translate into statistically significant improvements in overall clinical success, they suggest meaningful practical benefits in terms of treatment efficiency and healthcare utilization.

These findings support the use of metal stent-based approaches, particularly intraductal FCSEMS, as effective and less burdensome alternatives to conventional plastic stenting for the management of post-transplant anastomotic biliary strictures. Larger prospective multicenter studies are warranted to confirm these findings and further define the optimal endoscopic treatment strategy in this patient population.

## Figures and Tables

**Table 1:** Baseline Demographic and Transplant Characteristics.

Variable	Group 1 Plastic Stent (n = 32)	Group 2 FCSEMS (n = 30)	Group 3 Intraductal Metal Stent (n = 25)	*p*-Value
Age (years)	46.2 ± SD	55.2 ± SD	58.6 ± SD	<0.001
Male sex, *n* (%)	20 (62.5%)	14 (46.7%)	12 (48.0%)	0.39
MELD score	19.3	14.6	15.0	0.02
Right lobe graft, *n* (%)	31 (96.9%)	29 (96.7%)	23 (92.0%)	0.56
Left lobe graft, *n* (%)	1 (3.1%)	1 (3.3%)	2 (8.0%)	0.56
Hepatic artery thrombosis, *n* (%)	0 (0%)	0 (0%)	0 (0%)	NS
Portal vein complication, *n* (%)	2 (6.3%)	0 (0%)	2 (8.0%)	0.28
Cold ischemia time (min)	80.5	95.8	74.5	0.09
Hospital stay after LT (days)	16.4	13.0	14.9	0.18
PTK-assisted initial cannulation, *n* (%)	1 (3.1%)	1 (3.3%)	2 (8.0%)	0.629

Abbreviations: FCSEMS = Fully Covered Self-Expandable Metal Stent; LT = Liver Transplantation; NS = Non-significant.

**Table 2:** Indications for liver transplantation according to stent type.

Indication for Liver Transplantation	Plastic Stent (n = 32)	FCSEMS (n = 30)	ID-FCSEMS (n = 25)
HBV-related liver disease *	11 (34.4%)	10 (33.3%)	13 (52.0%)
Cryptogenic cirrhosis	7 (21.9%)	9 (30.0%)	3 (12.0%)
NASH	4 (12.5%)	5 (16.7%)	6 (24.0%)
Alcohol-related liver disease	3 (9.4%)	2 (6.7%)	0 (0%)
Autoimmune liver disease	1 (3.1%)	2 (6.7%)	2 (8.0%)
HCV-related liver disease	0 (0%)	0 (0%)	1 (4.0%)
HCC without underlying viral hepatitis	1 (3.1%)	0 (0%)	0 (0%)
Other indications †	5 (15.6%)	2 (6.7%)	0 (0%)

Abbreviations: FCSEMS, fully covered self-expandable metal stent; ID-FCSEMS, intraductal fully covered self-expandable metal stent; HBV, hepatitis B virus; HCV, hepatitis C virus; NASH, nonalcoholic steatohepatitis. Footnote: *: HBV-related liver disease includes HBV cirrhosis, HBV-associated hepatocellular carcinoma, HBV/HDV coinfection, and fulminant hepatitis B. †: Other indications include Budd–Chiari syndrome, Wilson disease, primary hyperoxaluria, familial cholestatic cirrhosis, and other rare indications for liver transplantation.

**Table 3:** Stricture-related and procedural characteristics according to initial stent strategy.

Variable	MPS (n = 32)	FCSEMS (n = 30)	ID-FCSEMS (n = 25)	*p* Value
Time from LT to stricture diagnosis, months	10 (8.8–12.0)	10 (6.3–12.0)	10 (7.0–12.0)	0.686
Anastomosis type				0.655
Single	20 (62.5%)	16 (53.3%)	16 (64.0%)	
Double	12 (37.5%)	13 (43.3%)	9 (36.0%)	
Other	0	1 (3.3%)	0	
Stricture length				0.622
Short	24 (75.0%)	21 (70.0%)	18 (72.0%)	
Intermediate	5 (15.6%)	8 (26.7%)	4 (16.0%)	
Long	3 (9.4%)	1 (3.3%)	3 (12.0%)	
Stricture severity				0.044
Moderate	21 (65.6%)	25 (83.3%)	13 (52.0%)	
Severe	11 (34.4%)	5 (16.7%)	12 (48.0%)	
Upstream biliary dilatation	10 (31.3%)	9 (30.0%)	9 (36.0%)	0.826
Prior bile leak	2 (6.3%)	0	2 (8.0%)	0.316
Previous ERCP	0	1 (3.3%)	1 (4.0%)	0.544
Treatment duration/stent dwell time, months	12 (12.0–14.8)	10 (9.0–12.0)	9 (8.0–10.5)	<0.001

Abbreviations: LT, liver transplantation; ERCP, endoscopic retrograde cholangiopancreatography; MPS, multiple plastic stents; FCSEMS, fully covered self-expandable metal stent; ID-FCSEMS, intraductal fully covered self-expandable metal stent. Continuous variables are presented as median (interquartile range, IQR) and compared using the Kruskal–Wallis test. Categorical variables are presented as n (%) and compared using the χ^2^ test or Fisher’s exact test, as appropriate.

**Table 4:** Technical Success and Clinical Outcomes.

Outcome	Plastic	FCSEMS	Intraductal	*p*-Value
Technical + clinical success	26 (81.3%)	25 (83.3%)	23 (92.0%)	0.501
Recurrence	6 (18.8%)	3 (10.0%)	3 (12.0%)	0.579
Median ERCP sessions	5	2	2	<0.001
Time to stricture resolution (months)	12	10	9	<0.001
Cumulative hospitalization during treatment (days)	7	4	3	<0.001

Abbreviations: ERCP = Endoscopic Retrograde Cholangiopancreatography.

**Table 5:** Procedure-Related Complications.

Complication	Plastic	FCSEMS	Intraductal	*p*-Value
Cholangitis	12 (37.5%)	10 (33.3%)	8 (32.0%)	0.898
Post-ERCP pancreatitis	2 (6.3%)	2 (6.7%)	2 (8.0%)	0.965
Bleeding	2 (6.3%)	3 (10.0%)	1 (4.0%)	0.671
Perforation	0 (0%)	0 (0%)	1 (4.0%)	0.285
ICU admission	9 (28.1%)	3 (10.0%)	3 (12.0%)	0.120
Mortality	2 (6.3%)	1 (3.3%)	0 (0%)	0.439
Stent migration	7 (21.9%)	7 (23.3%)	3 (12.0%)	0.525
PTK requirement	1 (3.1%)	1 (3.3%)	2 (8.0%)	0.629

**Table 6:** Predictors of Clinical Success (Univariate Logistic Regression).

Variable	Odds Ratio (OR)	95% CI	*p*-Value
FCSEMS vs. plastic stent	1.15	0.31–4.27	0.830
Intraductal vs. plastic stent	2.65	0.49–14.47	0.259
Age, per year	1.04	0.99–1.10	0.124
Male sex	4.62	1.17–18.20	0.029
MELD score, per point	0.91	0.84–0.97	0.007
Right lobe graft	6.55	0.83–51.36	0.074
Hepatic vein complication	0.16	0.01–2.81	0.212
Postoperative bleeding	0.23	0.03–1.55	0.132
Stent migration	1.40	0.28–6.99	0.683
Cholangitis	0.82	0.24–2.76	0.744
Post-ERCP pancreatitis	0.31	0.05–1.93	0.211
ICU requirement	1.17	0.23–5.93	0.848
ERCP session number	0.89	0.65–1.23	0.488
Cumulative hospitalization during treatment (days)	0.96	0.79–1.16	0.663

Abbreviations: OR, odds ratio; CI, confidence interval; FCSEMS, fully covered self-expandable metal stent; ERCP, endoscopic retrograde cholangiopancreatography; ICU, intensive care unit; MELD, Model for End-Stage Liver Disease.

**Table 7:** Adjusted comparison of clinical and procedural outcomes according to initial stent strategy.

Outcome	FCSEMS vs. MPS Adjusted Estimate (95% CI)	*p*	ID-FCSEMS vs. MPS Adjusted Estimate (95% CI)	*p*
Clinical success	aOR 0.55 (0.11–2.85)	0.480	aOR 1.26 (0.16–9.79)	0.828
Recurrence	aOR 0.41 (0.08–1.99)	0.267	aOR 0.47 (0.09–2.51)	0.378
Stent migration	aOR 0.96 (0.26–3.57)	0.957	aOR 0.46 (0.09–2.36)	0.351
ERCP sessions	β −3.10 (−3.64 to −2.55)	<0.001	β −2.82 (−3.40 to −2.23)	<0.001
Treatment duration (months)	β −2.71 (−4.30 to −1.12)	0.001	β −3.72 (−5.38 to −2.05)	<0.001
Cumulative hospitalization during treatment (days)	β −3.02 (−4.61 to −1.44)	<0.001	β −4.24 (−6.12 to −2.36)	<0.001
Follow-up duration (months)	β 2.04 (−17.93 to 22.01)	0.840	β 17.31 (−6.73 to 41.36)	0.156

Hint: Binary outcomes were analyzed using multivariable logistic regression and are presented as adjusted odds ratios (aORs). Continuous outcomes were analyzed using multivariable linear regression and are presented as β coefficients. All models were adjusted for recipient age and MELD score because these variables differed significantly among treatment groups at baseline. MPS was used as the reference group.

**Table 8:** Sensitivity analysis additionally adjusted for stricture severity.

Outcome	FCSEMS vs. MPS Adjusted Estimate (95% CI)	*p*	ID-FCSEMS vs. MPS Adjusted Estimate (95% CI)	*p*	Effect of Severe Stricture	*p*
ERCP sessions	β −3.05 (95% CI −3.58 to −2.52)	<0.001	β −2.82 (95% CI −3.39 to −2.24)	<0.001	β +0.29 (95% CI −0.46 to 1.05)	0.446
Treatment duration (months)	β −2.23 (95% CI −3.62 to −0.84)	0.002	β −3.63 (95% CI −5.17 to −2.09)	<0.001	β +1.63 (95% CI 0.45 to 2.81)	0.007
Cumulative hospitalization during treatment (days)	β −2.80 (95% CI −4.28 to −1.31)	<0.001	β −4.23 (95% CI −6.08 to −2.38)	<0.001	β +0.58 (95% CI −0.33 to 1.49)	0.212

## Data Availability

The data presented in this study are available from the corresponding author upon reasonable request.
